# Supplementary material for: Risk factors for sacrococcygeal pilonidal sinus: a systematic review and meta-analysis supplemented by genetic causal assessment
Source: Front Surg. 2026 Jan 7;12:1718589. doi: 10.3389/fsurg.2025.1718589 (PMC12819706; doi:10.3389/fsurg.2025.1718589)
Supplement: Supplementary file 2 [file Datasheet2.zip › Supplementary Data 2/MR_pipeline_p5e-8/leaveone_plots/ukb-d-1747_1.finngen_R12_L12_PILONIDALCYST.pdf]

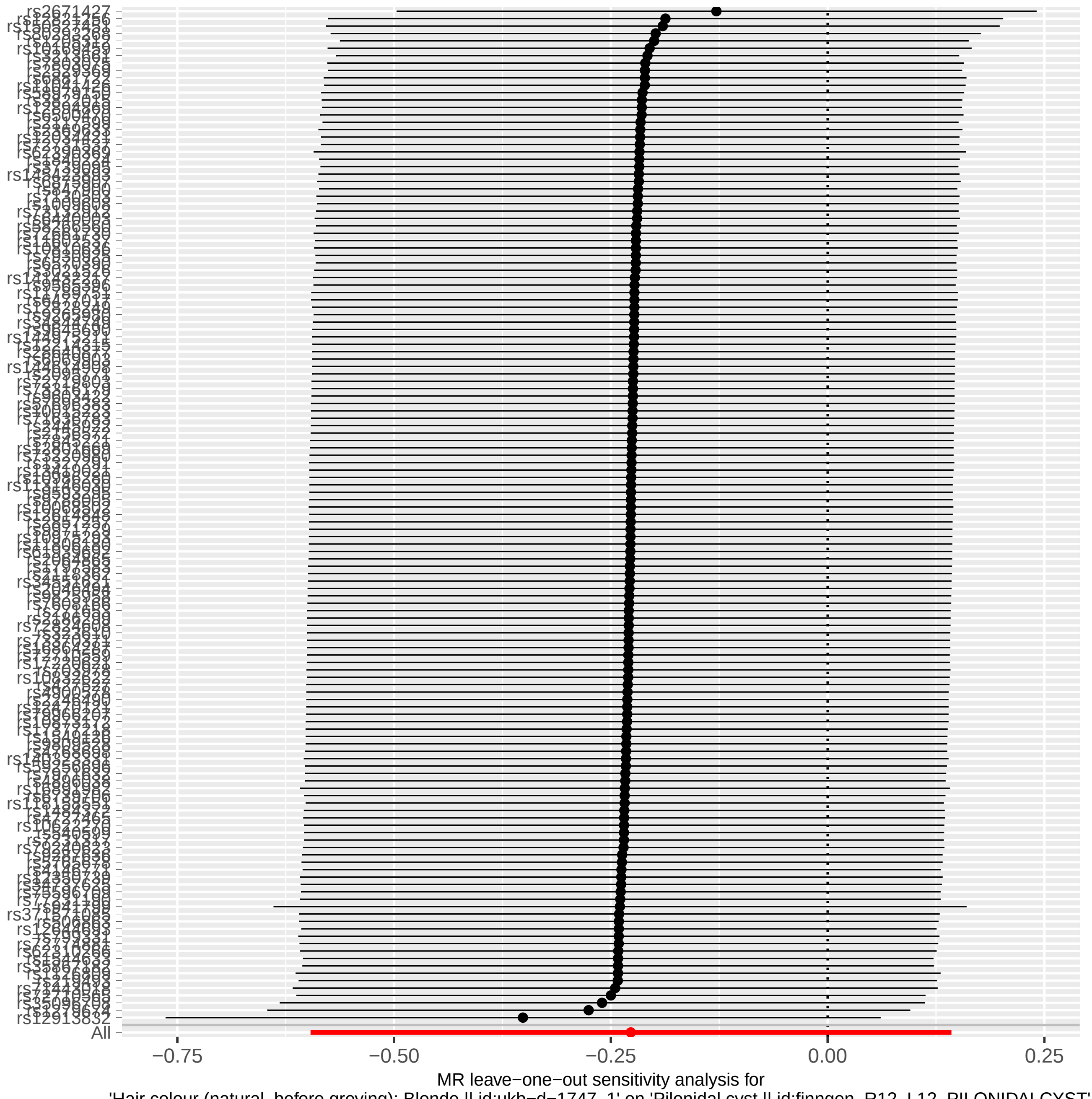
rs2671427
rs12913832
All
−0.75
−0.50
−0.25
0.00
0.25
MR leave−one−out sensitivity analysis for
'Hair colour (natural, before greying): Blonde || id:ukb−d−1747_1' on 'Pilonidal cyst || id:finngen_R12_L12_PILONIDALCYST'
